# Timing of onset of persistent critical illness in Japan: a nationwide registry study

**DOI:** 10.1016/j.lanwpc.2025.101632

**Published:** 2025-07-09

**Authors:** Hiroyuki Ohbe, Daisuke Kudo, Naoya Kobayashi, Kasumi Shirasaki, Kensuke Nakamura, Theodore J. Iwashyna, Shigeki Kushimoto

**Affiliations:** a Department of Emergency and Critical Care Medicine, Tohoku University Hospital, Miyagi, Japan; b Department of Clinical Epidemiology and Health Economics, School of Public Health, The University of Tokyo, Tokyo, Japan; c Division of Emergency and Critical Care Medicine, Tohoku University Graduate School of Medicine, Miyagi, Japan; d Department of Anesthesiology and Perioperative Medicine, Tohoku University Graduate School of Medicine, Miyagi, Japan; e Department of Emergency and Critical Care Medicine, St. Luke’s International Hospital, Tokyo, Japan; f Department of Emergency and Disaster Medicine, Kanazawa University Hospital, Ishikawa, Japan; g Department of Critical Care Medicine, Yokohama City University Hospital, Kanagawa, Japan; h Division of Pulmonary and Critical Care Medicine, Department of Medicine, Johns Hopkins University, Baltimore, MD, United States; i Department of Health Policy and Management, Bloomberg School of Public Health, Johns Hopkins University, Baltimore, MD, United States

**Keywords:** Persistent critical illness, ICU, End-of-life care, Critical care delivery, Japan

## Abstract

**Background:**

Persistent critical illness (PerCI) occurs in patients whose ongoing ICU stay is no longer primarily driven by their presenting illness, but rather by new clinical instability. Studies in Western countries have found PerCI onset at 5–11 days, but timing in Japan or other Asian countries is unknown.

**Methods:**

Using the Japanese Intensive Care Patient Database (JIPAD), PerCI onset was assessed by comparing the predictive ability of antecedent characteristics components (demographics, comorbidities) and acute illness components (physiological status at ICU admission) for in-hospital mortality, analyzed through multivariable logistic regression models among patients still in ICU from day 1 to day 28. The day when the Area Under the Receiver Operating Characteristic curve (AUROC) of antecedent characteristics exceeded that of acute illness was defined as the onset of PerCI.

**Findings:**

We studied 285,567 patients from 101 ICUs in Japan between April 2015 and March 2023. Overall in-hospital mortality was 8.2%. The AUROCs of antecedent characteristics and acute illness components for in-hospital mortality was 0.752 and 0.920 on day 1, 0.669 and 0.779 on day 7, 0.668 and 0.743 on day 14 and 0.667 and 0.700 on day 28, respectively. Through day 28, the AUROC of acute illness component remained consistently higher than that of antecedent characteristics component, with no observed onset of PerCI.

**Interpretation:**

Unlike findings from Western countries, PerCI onset was not observed in ICU in Japan. This suggests that variation in end-of-life decision making and the critical care delivery system may influence the onset of PerCI.

**Funding:**

None.

**Research in context**

**Evidence before this study**

To understand the existing knowledge on the timing of onset of persistent critical illness (PerCI) before conducting our study, we performed a comprehensive literature search. We searched databases including PubMed for studies published up to March 1, 2025, without language restrictions, using the search term “persistent critical illness”. 57 citations were identified and a total of 7 studies examined the timing of onset of PerCI. All studies were from Western developed countries and they have identified varying onset of PerCI, in Australia and New Zealand, this onset occurred at 10 days in the ICU. In a UK study it was at day 11, in Canada at day 9, in the Netherlands at day 7, in Scotland at day 5, and in the US Veteran Health Administration at day 16–24. The mechanism driving onset of persistent critical illness is unknown. There was a lack of data regarding the timing of onset of PerCI in Asian populations, particularly in Japan.

**Added value of this study**

To our knowledge, this is the first investigation into the timing of onset of PerCI in Japan, analyzing data from 285,567 ICU patients across 101 ICUs. Contrary to findings from Western developed countries, where PerCI onset has been observed on day 5 through 16 of ICU stay, PerCI onset was not observed within the first 28 days of ICU admission in this study. Since this study replicated the methodology of previous research, the absence of an observed PerCI onset in our study likely reflects differences in the pattern of data in this study compared to previous studies rather than difference in methodology or random variation.

**Implications of all the available evidence**

The absence of PerCI onset in the Japanese ICU population indicates that ICU practices, including end-of-life care and critical care delivery system, may influence the onset of PerCI. Furthermore, this implies that PerCI is modifiable and changes in ICU practice could avoid PerCI onset. Expanding international studies on PerCI could be highly impactful in identifying factors influencing PerCI’s timing of onset, contribute to a better understanding of its underlying mechanisms, and explore whether PerCI timing can serve as an indicator of end-of-life care practices within different healthcare systems.

## Introduction

Advances in critical care have prolonged survival among intensive care unit (ICU) patients, leading to the emergence of a new patient population—those who survive the initial acute phase of critical illness, but require prolonged ICU stays and develop chronic critical symptoms.^1^^,^^2^ Such patients (or subsets of them) are sometimes termed persistent critical illness (PerCI). The concept of PerCI was first proposed by Iwashyna et al., in 2015, and their conceptual definition of the timing of onset of PerCI was a group of patients whose reason for being in an ICU is no longer primarily driven by their presenting illness.^3^, ^4^, ^5^ This conceptual definition has been operationalized by defining PerCI onset as the time point when the predictive ability (e.g., Area Under the Receiver Operating Characteristic curve [AUROC]) of antecedent characteristics—such as demographics and comorbidities—for in-hospital mortality surpasses that of acute illness components, typically measured as physiological status at ICU admission.^5^ The clinical significance of identifying PerCI patients lies by focusing and intervening on other new reasons for ICU stay after acute illness resolution, which distinguish them from chronic critical illness and prolonged mechanical ventilation and so-called “failure to wean” patients.^3^^,^^4^ Patients with PerCI require higher ICU resources and have significant poorer outcomes.^5^

Timing of onset of PerCI have since been explored in Western developed countries. Studies have identified varying onset of PerCI, in Australia and New Zealand, this onset occurred at 10 days in the ICU.^5^^,^^6^ In a UK study it was at day 11,^7^ in Canada at day 9,^8^ in the Netherlands at day 7,^9^ in Scotland at day 5,^10^ and in the US Veteran Health Administration at day 16–24.^11^ While the US Veterans study reported a later transition point— perhaps due to differences in modeling approach (regression model with variable selection) and patient population (only 3.5% female)—,^11^ most studies have identified the transition within 5–11 days. This consistency has been interpreted as demonstrating that PerCI is often a physiologic process, potentially related to frailty and the inability to re-establish homeostasis.^12^ This interpretation has been challenged by other data from one health system suggesting an association with hospital quality which is not logically correlated with variation in patients’ physiologic reserve.^13^

Further adding to our knowledge gaps, studies to date have been limited to Western developed countries, leaving a significant knowledge gap regarding PerCI in Asia, including Japan. Given the possibility that PerCI onset is driven by differences in clinical practice, it is unclear whether the findings obtained in data from Western developed countries will be replicated in Asian countries, especially in Japan, which is the most aged country in the world.^14^^,^^15^

Therefore, this study aims to evaluate the timing, incidence, and healthcare burden of PerCI in Japan using a national ICU registry, based on the established framework of PerCI. We hypothesize that PerCI onset in Japan mirrors the 10 day transition point identified in Western developed countries, with comparable resource implications.

## Methods

### Study design and data source

This was a multicenter retrospective cohort study conducted in Japan. We used data from a clinical registry operated by the Japanese Society of Intensive Care Medicine, known as the Japanese Intensive Care Patient Database (JIPAD).^16^ JIPAD, the largest intensive care patient registry in Japan, began data collection in 2015 and included voluntary participating 101 ICUs as of March 2023. All patients registered in the JIPAD were admitted to ICUs, which maintain a nurse-to-patient ratio of one to two, and were subsequently discharged from the hospital. Participating ICUs were required to provide the JIPAD with data on all admitted patients. To ensure the validity and credibility of the data, the collected information is validated when ICUs begin to participate in the JIPAD and is subsequently monitored regularly by members of the JIPAD Working Group.^16^ The current study was approved by the Ethics Committee of Tohoku University Hospital, Japan, before the dataset was obtained (IRB approval No. 2024-1-1018, approved on March 26, 2025), following the Strengthening the Reporting of Observational Studies in Epidemiology (STROBE) guidelines. The need to collect informed consent was waived because all data were handled in an anonymized fashion.

### Population, exposure, and outcomes

We replicated the definitions of population, exposure, outcome, and statistical analysis from the studies performed by Iwashyna et al.^5^

All patients admitted to ICUs in the JIPAD between April 1, 2015, and March 31, 2023 were eligible for inclusion in this study. Exclusion criteria were patients younger than 15 years old, patients admitted to ICU solely for undergoing procedures, patients transferred from another hospital, patients with second or subsequent ICU admissions during the same hospitalization, patients whose hospitals data could not be combined, and patients with missing ICU admission and discharge dates.

### The primary outcome was in-hospital mortality

The exposure variables were risk-of-death scores consisting of two parts: (1) an antecedent characteristics component and (2) an acute illness component. The antecedent characteristics component included demographics (age, sex), comorbidities, hospital characteristics (hospital type, ICU volume), and temporal trends relating to the timing of ICU admission (hour, day, month, year). The acute illness component included admission diagnosis of 122 individual Acute Physiology and Chronic Health Evaluation (APACHE) III diagnoses, each of the 16 APACHE III acute physiology score, type of ICU admission (elective surgical, urgent surgical, medical), source of ICU admission (emergency department, operation room, general ward, other), pre-ICU hospital length of stay, invasive mechanical ventilation within first 24 h of ICU admission, medical emergency team call before ICU admission, and cardiac arrest before ICU admission. All acute illness component could be collected within first 24 h of ICU admission. Where continuous physiological variables were missing, we did single imputation with normal value substitution in accordance with standard clinical risk modelling.^17^

### Statistical analysis

Multivariable analyses for in-hospital mortality were performed using logistic regression, with separate models developed each day among patients still in ICU from day 1 (i.e., the first 24 h of ICU admission) to day 28. These analyses incorporated three distinct models: one for the antecedent characteristics component, another for the acute illness component, and, for stratification by initial risk of death, a third for combination of both antecedent characteristics component and acute illness component. The contributions of antecedent characteristics component and acute illness component to in-hospital mortality prediction were assessed using differences in the AUROC. Consistent with previous studies,^5^, ^6^, ^7^, ^8^, ^9^, ^10^, ^11^ the timing of onset of PerCI was defined as the first day where the point estimate of AUROC for the models of acute characteristics component became lower than that for antecedent characteristics component.

Subgroup analyses were performed for (a) age (15–64 years, 65–74 years, 75 years and older), (b) type of ICU admission (medical, elective surgical, urgent surgical), (c) fiscal year 2015–2019 and 2020–2022 before and after COVID-19 pandemic, (d) admission diagnosis (cardiac surgery, other cardiovascular, gastrointestinal, and respiratory).

As a sensitivity analysis, we repeated the primary analysis after excluding day of the week and time of day from the antecedent characteristics component.

To evaluate mortality trajectories stratified by patients’ overall probability of death on ICU admission, patients were categorized into three groups: low (<33%), moderate (33–66%), and high (>66%). Probability of death is based on their ICU admission total predicted risk of death from a logistic regression including both antecedent characteristics and acute illness component. Odds of in-hospital death for patients with high and moderate probability of death vs. those with low probability of death were calculated, adjusted for differences between groups in antecedent characteristics component.

All analyses were performed using Stata/MP 17.0 software (StataCorp). All reported *P* values are two-sided, and *P* < 0.05 was considered statistically significant. Missing continuous physiological variables were imputed using single imputation with normal value in accordance with standard clinical risk modeling practices.^17^ Missingness for physiological variables were shown in Supplementary Table S1, in all variables less than 10%, and in 11 of 16 variables less than 2%.

## Results

Between April 1, 2015, and March 31, 2023, there were 324,473 patients admitted to the 101 ICUs registered in the JIPAD. After exclusion, a total of 285,567 patients (88%) admitted to 101 ICUs remained eligible in this study (Supplementary Fig. S1).

The median age of the cohort was 71 years (interquartile range 59–78), and 61.3% were male (Table 1). More than half of ICU admissions were elective surgery (58.5%), followed by medical (28.7%) and emergency surgery (12.8%). Of 285,567 eligible patients, 153,110 (53.6%) remained in the ICU on day 2, 35,651 (12.5%) on day 7, 20,478 (7.2%) on day 10, 11,508 (4.0%) on day 14, 4709 (1.6%) on day 21, and 2551 (0.9%) on day 28. Overall, in-hospital mortality was 8.2% (n = 23,307/285,567). In-hospital mortality increased progressively for patients remaining in the ICU, 11.3% on day 2, 22.8% on day 7, 28.5% on day 10, 33.4% on day 14, 42.3% on day 21, and 46.7% on day 28.

**Table 1:** Demographic characteristics and outcomes of ICU patients.

Variables	Patients still in ICU						
	Day 1 (Overall)	Day 2	Day 7	Day 10	Day 14	Day 21	Day 28
Total number of patients in cohort	N = 285,567	N = 153,110	N = 35,651	N = 20,478	N = 11,508	N = 4709	N = 2551
% of overall patients	100%	53.6%	12.5%	7.2%	4.0%	1.6%	0.9%
Fiscal year							
2015–2019	118,079 (41.3%)	60,541 (39.5%)	13,509 (37.9%)	7620 (37.2%)	4264 (37.1%)	1716 (36.4%)	917 (35.9%)
2020–2022	167,488 (58.7%)	92,569 (60.5%)	22,142 (62.1%)	12,858 (62.8%)	7244 (62.9%)	2993 (63.6%)	1634 (64.1%)
Age (years)	71 (59–78)	71 (59–78)	71 (58–78)	70 (58–78)	70 (57–78)	69 (57–77)	69 (57–77)
Age category							
15–64	96,986 (34.0%)	51,288 (33.5%)	12,447 (34.9%)	7308 (35.7%)	4248 (36.9%)	1762 (37.4%)	977 (38.3%)
65–74	84,046 (29.4%)	44,217 (28.9%)	9885 (27.7%)	5679 (27.7%)	3158 (27.4%)	1343 (28.5%)	749 (29.4%)
75–	104,535 (36.6%)	57,605 (37.6%)	13,319 (37.4%)	7491 (36.6%)	4102 (35.6%)	1604 (34.1%)	825 (32.3%)
Male sex	174,988 (61.3%)	97,015 (63.4%)	23,030 (64.6%)	13,205 (64.5%)	7438 (64.6%)	3117 (66.2%)	1685 (66.1%)
APACHE III score on admission	52 (39–70)	62 (47–80)	78 (61–100)	83 (64–105)	84 (66–107)	85 (67–108)	84 (67–107)
APACHE III risk of death	0.07 (0.03–0.20)	0.12 (0.05–0.33)	0.32 (0.13–0.62)	0.38 (0.16–0.67)	0.40 (0.17–0.69)	0.40 (0.18–0.70)	0.39 (0.17–0.69)
Type of admission							
Elective surgery	167,015 (58.5%)	61,139 (39.9%)	6667 (18.7%)	2863 (14.0%)	1676 (14.6%)	795 (16.9%)	493 (19.3%)
Emergency surgery	36,528 (12.8%)	27,884 (18.2%)	9031 (25.3%)	5432 (26.5%)	3034 (26.4%)	1093 (23.2%)	558 (21.9%)
Medical	82,024 (28.7%)	64,087 (41.9%)	19,953 (56.0%)	12,183 (59.5%)	6798 (59.1%)	2821 (59.9%)	1500 (58.8%)
Major diagnostic category							
Cardiac surgery	31,830 (11.1%)	24,027 (15.7%)	6439 (18.1%)	3649 (17.8%)	1926 (16.7%)	746 (15.8%)	370 (14.5%)
Other cardiovascular	40,863 (14.3%)	18,935 (12.4%)	6580 (18.5%)	4266 (20.8%)	2517 (21.9%)	1159 (24.6%)	642 (25.2%)
Gastrointestinal	57,796 (20.2%)	24,468 (16.0%)	4450 (12.5%)	2384 (11.6%)	1334 (11.6%)	565 (12.0%)	298 (11.7%)
Respiratory	66,305 (23.2%)	43,594 (28.5%)	7915 (22.2%)	3885 (19.0%)	2282 (19.8%)	1125 (23.9%)	677 (26.5%)
Mechanical ventilation in first 24 h	102,965 (36.1%)	85,741 (56.0%)	27,109 (76.0%)	16,178 (79.0%)	9271 (80.6%)	3801 (80.7%)	2053 (80.5%)
RRT in the ICU	21,658 (7.6%)	19,196 (12.5%)	9045 (25.4%)	6292 (30.7%)	4206 (36.5%)	2251 (47.8%)	1348 (52.8%)
ECMO in the ICU	3502 (1.2%)	2982 (1.9%)	2205 (6.2%)	1765 (8.6%)	1322 (11.5%)	785 (16.7%)	510 (20.0%)
ICU mortality	10,975 (3.8%)	7008 (4.6%)	3351 (9.4%)	2571 (12.6%)	1840 (16.0%)	1122 (23.8%)	731 (28.7%)
In-hospital mortality	23,307 (8.2%)	17,279 (11.3%)	8142 (22.8%)	5842 (28.5%)	3843 (33.4%)	1991 (42.3%)	1191 (46.7%)
Duration of ICU stay (days)	2 (1–4)	3 (2–6)	10 (8–15)	14 (11–20)	19 (15–26)	28 (24–38)	37 (31–49)
Duration of hospital stay (days)	20 (12–35)	25 (15–43)	41 (25–67)	48 (30–78)	56 (35–89)	68 (43–107)	79 (50–125)
Discharge destination							
Home	213,338 (74.7%)	99,994 (65.3%)	14,090 (39.5%)	6157 (30.1%)	2852 (24.8%)	927 (19.7%)	438 (17.2%)
Other hospital	48,922 (17.1%)	35,837 (23.4%)	13,419 (37.6%)	8479 (41.4%)	4813 (41.8%)	1791 (38.0%)	922 (36.1%)

Data are presented as median (interquartile range) and number (percentage) as appropriate. Primary demographic characteristics and outcomes are summarized in this table, while additional variables are provided in the Supplemental Tables S3 and S4.

ICU, intensive care unit; APACHE, Acute Physiology and Chronic Health Evaluation; RRT, renal replacement therapy; ECMO, extracorporeal membrane oxygenation.

**Fig. 1:**
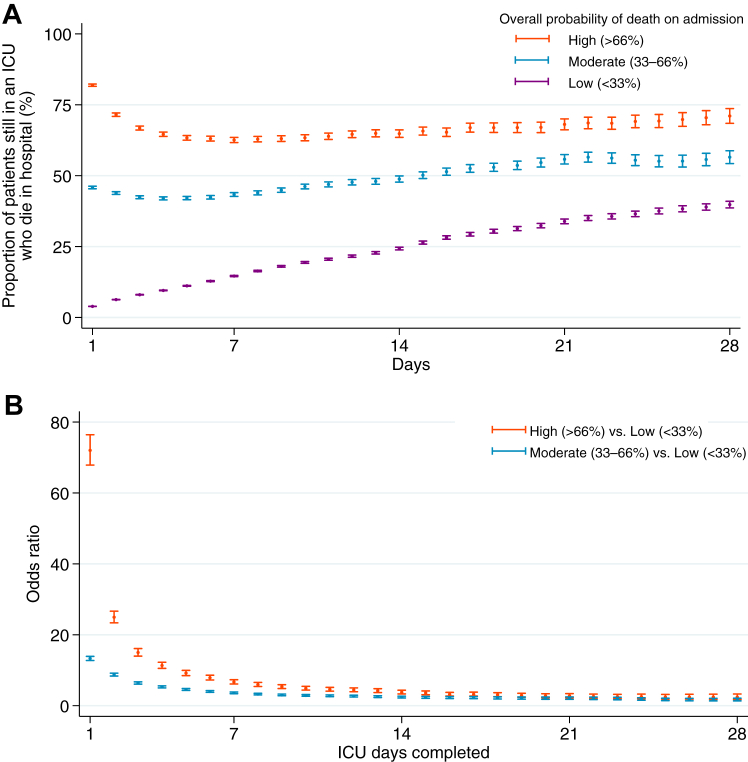
Mortality for patients in an ICU. (A) Unadjusted in-hospital mortality, stratified by patients’ overall probability of death on ICU admission. Probability of death is based on their ICU admission total predicted risk of death from a logistic regression including both antecedent characteristics and acute illness component. Error bars are SEs. (B) Odds of in-hospital death for patients with high and moderate probability of death on admission vs. those with low probability of death of those still in an ICU on a given day. This statistic is adjusted for differences between groups in antecedent characteristics component. Error bars are 95% CIs. The numbers of patients between groups are unbalanced as they were chosen for illustrative purposes to span clinically relevant ranges of patients. We chose these cutpoints dividing low (<33%), moderate (33–66%), and high (>66%) probability of death to divide up the continuous distribution of risk of following previous study conducted by Iwashyna et al. ICU, intensive care unit.

Among the patients with a low likelihood of death on admission (predicted mortality of <33% on the basis of their day 1 total predicted risk of death from both combined acute and antecedent models), the unadjusted in-hospital mortality increased steadily from 3.9% on day 1–39.8% on day 28 (Fig. 1 and Supplementary Table S2). By contrast, among patients who had a high likelihood of death on admission (predicted mortality of >66% on admission), the unadjusted in-hospital mortality decreased from 81.9% on day 1 to its lowest point of 62.6% on day 7, then increased again, following a U-shaped pattern, reaching 71.1% by day 28. Patients with the high likelihood of death had a 72.0-times greater odds of death on ICU admission than did those with the low likelihood of death (Fig. 1) after adjustment for antecedent characteristics components. These odds ratios declined rapidly until about day 10 (4.90-times greater odds of death), followed by a gradual decline, with limited convergence by day 28 (2.41-times greater odds of death).

**Fig. 2:**
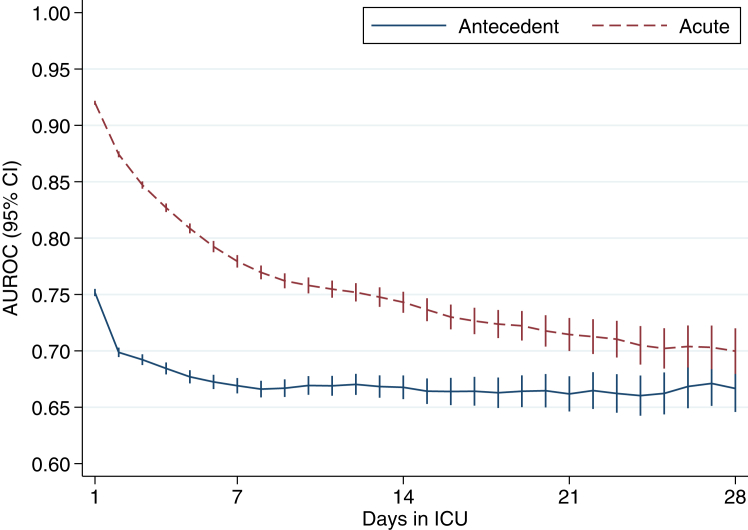
Predictive ability (measured by AUROC) for in-hospital mortality in the models of antecedent characteristics and acute illness component. Vertical bars are 95% CIs. The Supplemental Tables S3 and S4 contains all characteristics and regression weights (β coefficients from derivation sample). AUROC, area under the receiver operating characteristics; CI, confidence interval; ICU, intensive care unit.

Fig. 2 and Table 2 shows the AUROC for the models of antecedent characteristics component and acute illness component. On day 1, the predictive ability of antecedent model was moderate, with an AUROC of 0.752 (95% confidence interval [CI] 0.749–0.755), while that of acute model was high, with an AUROC of 0.920 (95% CI 0.918–0.922). The AUROC for the models of antecedent characteristics component reached its nadir around day 7 (AUROC 0.669) and plateaued thereafter, whereas that of acute illness component gradually diminished until day 28. On day 10, identified in previous studies as the onset of PerCI,^5^^,^^6^ the point estimate of AUROC of antecedent characteristics component and acute illness component were 0.669 and 0.758, respectively. By day 28, the AUROC of acute illness component remained consistently superior to that of antecedent characteristics component, with point estimate of AUROCs on day 28 of 0.667 and 0.700, respectively, and no onset of PerCI was observed, even when taking into account the uncertainty in estimation of the AUROCs. On the day of ICU admission, the acute illness component added significant predictive ability by combining with antecedent characteristics component (AUROC increase of 0.174), and this difference gradually narrowed to 0.105 on day 10 and remained at 0.071 on day 28, but did not disappear entirely (Table 2). The Supplementary Table S3 and S4 contains all antecedent characteristics component and acute illness component and their regression weights of the model.

**Table 2:** Predictive ability (measured by AUROC) for in-hospital mortality in the models of antecedent characteristics component, acute illness component, and combination of both antecedent characteristics component and acute illness component.

ICU day	Number in cohort	Antecedent component, AUROC (95%CI)	Acute illness component, AUROC (95%CI)	Combined model, AUROC (95%CI)	Antecedent vs. Combine
1	285,567	0.752 (0.749–0.755)	0.920 (0.918–0.922)	0.926 (0.924–0.928)	0.174
2	153,110	0.699 (0.694–0.703)	0.874 (0.872–0.877)	0.883 (0.881–0.886)	0.184
3	103,021	0.692 (0.687–0.697)	0.847 (0.844–0.850)	0.857 (0.854–0.860)	0.165
4	75,362	0.684 (0.679–0.690)	0.827 (0.823–0.831)	0.839 (0.835–0.842)	0.154
5	56,918	0.677 (0.671–0.683)	0.809 (0.804–0.813)	0.822 (0.817–0.826)	0.144
6	44,637	0.672 (0.666–0.679)	0.792 (0.788–0.797)	0.806 (0.801–0.811)	0.134
7	35,651	0.669 (0.662–0.676)	0.779 (0.774–0.785)	0.794 (0.789–0.800)	0.125
8	28,821	0.666 (0.659–0.673)	0.770 (0.764–0.776)	0.785 (0.779–0.791)	0.119
9	24,087	0.667 (0.659–0.675)	0.762 (0.756–0.769)	0.778 (0.771–0.784)	0.111
10	20,478	0.669 (0.661–0.678)	0.758 (0.751–0.765)	0.775 (0.768–0.782)	0.105
11	17,637	0.669 (0.660–0.678)	0.755 (0.747–0.762)	0.772 (0.765–0.779)	0.103
12	15,313	0.670 (0.661–0.680)	0.752 (0.744–0.760)	0.770 (0.762–0.778)	0.100
13	13,338	0.668 (0.658–0.678)	0.748 (0.739–0.756)	0.766 (0.758–0.775)	0.098
14	11,508	0.668 (0.657–0.678)	0.743 (0.734–0.752)	0.763 (0.754–0.772)	0.095
15	9643	0.664 (0.653–0.676)	0.737 (0.726–0.747)	0.757 (0.747–0.767)	0.093
16	8302	0.664 (0.652–0.676)	0.730 (0.719–0.741)	0.752 (0.741–0.763)	0.088
17	7317	0.664 (0.651–0.677)	0.727 (0.715–0.738)	0.749 (0.738–0.760)	0.085
18	6488	0.663 (0.649–0.676)	0.724 (0.711–0.736)	0.748 (0.736–0.760)	0.085
19	5861	0.664 (0.650–0.678)	0.722 (0.709–0.735)	0.747 (0.734–0.759)	0.083
20	5261	0.665 (0.650–0.679)	0.718 (0.704–0.732)	0.744 (0.731–0.757)	0.079
21	4709	0.662 (0.646–0.677)	0.715 (0.700–0.729)	0.742 (0.728–0.756)	0.080
22	4253	0.665 (0.649–0.681)	0.713 (0.697–0.728)	0.742 (0.727–0.757)	0.077
23	3860	0.662 (0.645–0.679)	0.710 (0.694–0.727)	0.741 (0.725–0.756)	0.078
24	3557	0.660 (0.643–0.678)	0.705 (0.688–0.722)	0.735 (0.719–0.752)	0.075
25	3257	0.662 (0.644–0.681)	0.702 (0.684–0.720)	0.734 (0.717–0.752)	0.072
26	2992	0.668 (0.649–0.688)	0.704 (0.685–0.722)	0.739 (0.721–0.756)	0.070
27	2767	0.671 (0.651–0.691)	0.703 (0.684–0.722)	0.740 (0.722–0.759)	0.069
28	2551	0.667 (0.646–0.687)	0.700 (0.680–0.720)	0.738 (0.719–0.757)	0.071

AUROC, area under the receiver operating characteristics; CI, confidence interval; ICU, intensive care unit.

Across all age groups in the subgroup analyses, AUROC of acute illness component remained consistently superior to that of antecedent characteristics component, with no observed onset of PerCI, consistent with the main analysis (Table 3 and Supplementary Fig. S2). Similarly, medical patients and patients in the fiscal year 2020–2022 showed no onset of PerCI. In the elective surgery patients, AUROC of acute illness component became lower than that of the antecedent characteristics component at day 20, with 0.5% (n = 841/167,015) in the cohort at that time. In the major diagnostic category stratified analysis—which represents a form of strong control for some acute illness components and so may not be directly comparable to the whole population analysis—the onset of PerCI varied by condition: cardiac surgery at day 24, other cardiovascular at day 17, gastrointestinal at day 20, and respiratory at day 13, with incidence ranging from 0.5% to 4.2% of the cohort (Table 3 and Supplementary Fig. S3). In these subgroup analyses where the onset of PerCI was observed, the AUROC of the antecedent characteristics component plateaued once and then increased, leading to a crossover with that of acute illness component.

**Table 3:** The timing of onset of PerCI.

Cohort	Number in cohort, n (%)	In-hospital Mortality, n (%)	Timing of onset of PerCI, days	Number of PerCI, n (%)
Overall	285,567 (100.0%)	23,307 (8.2%)	Not observed	Not applicable
**Subgroups**				
Age category				
15–64	96,986 (34.0%)	5918 (6.1%)	Not observed	Not applicable
65–74	84,046 (29.4%)	6082 (7.2%)	Not observed	Not applicable
75–	104,535 (36.6%)	11,307 (10.8%)	Not observed	Not applicable
Type of admission				
Elective surgery	167,015 (58.5%)	2201 (1.3%)	20	841 (0.5%)
Emergency surgery	36,528 (12.8%)	4095 (11.2%)	28	519 (1.4%)
Medical	82,024 (28.7%)	17,011 (20.7%)	Not observed	Not applicable
Fiscal year				
2015–2019	118,079 (41.3%)	9185 (7.8%)	24	1388 (1.2%)
2020–2022	167,488 (58.7%)	14,122 (8.4%)	Not observed	Not applicable
Major diagnostic category				
Cardiac surgery	31,830 (11.1%)	6451 (20.3%)	24	518 (1.6%)
Other cardiovascular	40,863 (14.3%)	4385 (10.7%)	17	1699 (4.2%)
Gastrointestinal	57,796 (20.2%)	3512 (6.1%)	20	610 (1.1%)
Respiratory	66,305 (23.2%)	2062 (3.1%)	13	2573 (3.9%)

The timing of onset of PerCI was defined as the first day where the point estimate of AUROC for the models of acute characteristics component became lower than that for antecedent characteristics component.

PerCI, persistent critical illness; AUROC, area under the receiver operating characteristics.

The results of sensitivity analysis excluding day of the week and time of day from the antecedent characteristics model showed no meaningful change in the main, with no observed onset of PerCI (Supplementary Fig. S4).

## Discussion

This study examined the timing of onset of PerCI using JIPAD data from Japan of 285,567 patients admitted to 101 ICUs from April 2015 to March 2023. Contrary to findings from Western developed countries, where PerCI onset has been observed on day 5 through 16 of ICU stay,^5^, ^6^, ^7^, ^8^, ^9^, ^10^, ^11^ this study did not identify a distinct crossover of AUROCs between antecedent and acute illness components in our cohort. Since this study replicated the methodology of previous research,^5^ with a large sample size that was representative of Japanese ICU population, the absence of an observed PerCI onset in our study likely reflects differences in the pattern of data in this study compared to previous studies rather than difference in methodology or random variation.

The trajectory of AUROC of the antecedent characteristics component was similar to that observed in previous studies, plateauing around 0.65–0.68.^5^, ^6^, ^7^, ^8^, ^9^, ^10^, ^11^ However, the key difference from previous studies lay in the trajectory of AUROC of the acute illness component, which remained high and was still above 0.70 even at day 28. Statistically, this suggests that patients for which the acute illness component alone predicts a high likelihood of in-hospital death continued to die at increased rates much longer than they did in Western ICUs.

We believe these data suggest that an interpretation of PerCI as representing primarily an irretrievable physiologic state of frailty may not be a complete answer, and support instead interpretations that health system variation in practice may drive the onset of PerCI—implying that it is modifiable, and if inconsistent with patient values, then changes in practice could promote recovery from PerCI or transition to alternative goals of care.

One of the primary factors that may contribute to this phenomenon is variation in end-of-life care practices in the ICU in Japan relative to many Western nations.^14^ In Western developed countries, death in the ICU often results from cessation of resuscitative efforts and life support, rather than purely physiologic causes without treatment limitations.^18^ Additionally, time-limited trials and earlier integration of palliative care and hospice are increasingly available.^19^^,^^20^ In contrast, cessation of resuscitative efforts and life support is rarely performed in the ICU in Japan due to the uncertain and threatening environment where withholding or withdrawal of care might lead to criminal prosecution.^14^ Additionally, in Japan, physicians are less likely to discuss treatment plans with nurses and more likely to continue full support for patients in vegetative states who develop complications compared to practices in Western developed countries.^21^^,^^22^ These practices align with trends observed in other East Asian countries such as China and South Korea, where Confucian values emphasize family-centered decision-making, respect for elders, and a reluctance to withdraw life-sustaining treatment, often leading to extended futile critical care.^23^, ^24^, ^25^

Another contributing factor is the difficulty in transitioning patients from the ICU to palliative care or intermediate care unit in Japan for those requiring life support with low or no possibility of survival, as described above. The Society of Critical Care Medicine’s guidelines for ICU admission and discharge recommend that such patients receive care in intermediate care units or palliative care settings rather than in the ICU.^26^ However, in Japan, palliative care units are specialized wards dedicated to cancer patients and do not function providing care for critically ill patients.^27^ Additionally, as of 2022, about 30% of ICU beds in Japan were located in hospitals without intermediate care unit beds.^28^ Furthermore, Japan’s ICU admission criteria are influenced by financial incentives tied to monitoring and procedures, meaning that even patients with no chance of survival, for whom treatment has been withheld, may remain in the ICU simply because hospitals can bill ICU-level costs, creating an additional incentive for ICU stays.^29^ Since April 2022, ICU reimbursement in Japan can be extended up to 25 days for patients receiving acute blood purification therapy or extracorporeal membrane oxygenation, and up to 30 days for organ transplant patients, compared with the standard limit of 14 days for other ICU admissions. Similarly, a previous study in Scotland showed that increased ICU capacity was associated with delayed onset of PerCI,^10^ suggesting evidence that critical care delivery system may influence the timing of onset of PerCI.

However, we acknowledge that the exact mechanisms by which such health system and cultural differences could prevent or obscure the typical transition to persistent critical illness remain uncertain. While we propose several plausible hypotheses—including differences in end-of-life decision making, ICU discharge criteria, medical insurance system, medico-economical factor, and the availability of step-down or intermediate care—these explanations remain speculative. Further research, including international collaborative studies, will be essential to directly clarify how institutional and sociocultural factors influence the epidemiology or recognition of persistent critical illness.

Subgroup analyses identified an onset of PerCI in some populations, but these findings must be interpreted with caution. The number of patients decreases as ICU days progress, and sample sizes in these subgroup analyses may be insufficient; moreover, reason for admission is a major acute illness component, and so stratification by reason for admission may limit the potential explanatory power of acute illness components. Notably, confidence intervals for AUROC estimates were wide, and the confidence intervals for antecedent and acute models overlapped. Additionally, in elective surgery, other cardiovascular, gastrointestinal, and respiratory subgroups, the AUROC of antecedent characteristics began increasing again around day 10. This suggests that proportion of patients who are highly likely to die based on antecedent characteristics (e.g., very elderly patients or cancer patients) increased in the patient population with a longer ICU stay. This pattern of antecedent AUROC rebound has not been observed in previous studies in Western developed countries and may suggest that a distinctive end-of-life care practices of low discrimination by antecedent characteristics in the ICU in Japan and East Asia, as described earlier.^23^, ^24^, ^25^

These findings have some important implications. First, the timing of onset of PerCI appears to be significantly influenced by sociocultural factors related to end-of-life care and the critical care delivery system. A recent scoping review on PerCI found that 15 out of 18 (83%) studies applied the ICU day 10 threshold based on the study by Iwashyna et al.^5^ Therefore, caution is warranted when applying this threshold without reassessing its applicability to different populations. Second, as this is the first study from Japan and Asia to examine the timing of onset of PerCI, the findings highlight the need for further investigations into timing of onset of PerCI in other Asian countries and previously unexamined regions. Third, our paradox results may present new challenges for future research on PerCI. Expanding international studies on PerCI could be highly impactful in identifying factors influencing PerCI’s timing of onset, contribute to a better understanding of its underlying mechanisms, and explore whether PerCI timing can serve as an indicator of end-of-life care practices within different healthcare systems. At the same time, our findings do not necessarily mean that chronically critically ill patients after the acute phase are absent in Japan. Rather, they may reflect limitations of the current understanding of PerCI, which may not fully capture the clinical reality in Japanese ICUs. Future studies may help to refine both the conceptual and operational definitions of PerCI, making them more broadly applicable across different healthcare systems and cultural contexts.

This study has several limitations. First, although we replicated prior research methodologies,^5^ we were unable to collect data on ICU admission sources (home vs. chronic care facility) and treatment limitations at ICU admission. However, both factors are part of antecedent characteristics component and given that the primary difference in our findings lies in the acute illness component, these variables are unlikely to have influenced our research. Although frailty data were not available in our registry, previous studies on PerCI onset timing have not included frailty in their primary models, and our study focused on timing rather than risk factors. Second, while the JIPAD is a national ICU registry, participation is voluntary. Therefore, there is a potential for selection bias, and the findings of this study may not be fully representative of all ICUs across Japan. Finally, the JIPAD was unable to identify patients who were provided end-of-life care during ICU stay. Therefore, we were unable to analyze what specific factors influence the timing of the onset of PerCI in this study. Future international studies are needed to elucidate these mechanisms.

### Conclusion

This study is the first to examine the timing of onset of PerCI in Japan using a national ICU registry, revealing no onset of PerCI as seen in Western developed countries. This suggests that ICU practices, including end-of-life care practices and critical care delivery system influence the onset of PerCI, and caution is warranted when applying the ICU day 10 threshold identified in previous PerCI study to Asian populations. Further research is needed to explore regional differences and mechanism of PerCI onset.

## Contributors

HO: Conceptualization, Methodology, Formal analysis, Data curation, Investigation, Writing—original draft, Writing—review & editing.

DK: Investigation, Writing—original draft, Writing—review & editing.

NK: Formal analysis, Data curation, Investigation, Writing—review & editing.

KS: Writing—original draft, Writing—review & editing.

KN: Writing—original draft, Writing—review & editing.

TI: Methodology, Writing—original draft, Writing—review & editing.

SK: Writing—review & editing, Supervision.

HO and NK accessed and verified the underlying data. HO had final responsibility for the decision to submit the manuscript. All authors contributed to the interpretation of results, revised the manuscript critically for important intellectual content, and approved the final version.

## Data sharing statement

The authors’ agreement with the JIPAD project does not allow us to publish the data used for this manuscript or to share it with others. The JIPAD Working Group would cooperate in case fraud or forgery is suspected on manuscripts in which JIPAD data is used.

## Declaration of interests

The authors do not have any competing interests.
