# Supplementary material for: Timing of onset of persistent critical illness in Japan: a nationwide registry study
Source: Lancet Reg Health West Pac. 2025 Jul 9;60:101632. doi: 10.1016/j.lanwpc.2025.101632 (PMC12274935; doi:10.1016/j.lanwpc.2025.101632)
Supplement: Supplementary Figs. S1–S4 and Tables S1–S4 [file mmc1.docx]

**Additional Files**

**Timing of onset of persistent critical illness in Japan: a nationwide registry study**

Hiroyuki Ohbe, Daisuke Kudo, Naoya Kobayashi, Kasumi Shirasaki, Kensuke Nakamura, Theodore J Iwashyna, Shigeki Kushimoto

**List of additional files**

**Supplementary Table 1.** Missingness for physiological variables.

**Supplementary Table 2.** Mortality for patients in an ICU.

**Supplementary Table 3.** Regression coefficients for antecedent characteristics component.

**Supplementary Table 4.** Regression coefficients for acute illness component.

**Supplementary Figure 1.** Patient flow chart.

**Supplementary Figure 2.** Predictive ability (measured by AUROC) for in-hospital mortality in the models of antecedent characteristics and acute illness component in the subgroup analyses for age and type of ICU admission.

**Supplementary Figure 3.** Predictive ability (measured by AUROC) for in-hospital mortality in the models of antecedent characteristics and acute illness component in the subgroup analyses for fiscal year and admission diagnosis.

**Supplementary Figure 4.** Predictive ability (measured by AUROC) for in-hospital mortality in the models of antecedent characteristics and acute illness component in the sensitivity analysis excluding day of the week and time of day from the antecedent characteristics model.

**Supplementary Table 1.** Missingness for physiological variables.

| Variable | Available, n | Missing, n (%) |
| --- | --- | --- |
| Heart rate | 284908 | 659 (0.2%) |
| Mean arterial pressure | 285307 | 260 (0.1%) |
| Temperature | 283991 | 1,576 (0.6%) |
| Respiratory rate | 284649 | 918 (0.3%) |
| PaO2 | 258184 | 27,383 (9.6%) |
| Hematocrit | 281105 | 4,462 (1.6%) |
| White cell count | 281456 | 4,111 (1.4%) |
| Creatinine | 280937 | 4,630 (1.6%) |
| Urine output | 283821 | 1,746 (0.6%) |
| Urea | 280020 | 5,547 (1.9%) |
| Sodium | 282615 | 2,952 (1.0%) |
| Albumin | 259395 | 26,172 (9.2%) |
| Bilirubin | 277605 | 7,962 (2.8%) |
| Glucose | 277612 | 7,955 (2.8%) |
| pH | 258175 | 27,392 (9.6%) |
| Glasgow Coma Scale | 285393 | 174 (0.1%) |

**Supplementary Table 2.** Mortality for patients in an ICU.

| Probability |  | Patients |  |  |  |  |
| --- | --- | --- | --- | --- | --- | --- |
| of | ICU | still in | Died in | In-hospital | Standard | Odds ratios |
| death | day | an ICU | hospital | mortality | error | (95% CI) |
| **High (>66%)** |  |  |  |  |  |  |
|  | 1 | 9149 | 7494 | 81.9% | 0.40% | 72.0 (67.9, 76.4) |
|  | 2 | 5549 | 3968 | 71.5% | 0.61% | 25.0 (23.4, 26.7) |
|  | 3 | 4541 | 3033 | 66.8% | 0.70% | 15.0 (14.0, 16.1) |
|  | 4 | 3947 | 2550 | 64.6% | 0.76% | 11.4 (10.5, 12.2) |
|  | 5 | 3507 | 2221 | 63.3% | 0.81% | 9.18 (8.47, 9.95) |
|  | 6 | 3152 | 1988 | 63.1% | 0.86% | 7.89 (7.24, 8.6) |
|  | 7 | 2833 | 1773 | 62.6% | 0.91% | 6.69 (6.11, 7.32) |
|  | 8 | 2532 | 1592 | 62.9% | 0.96% | 5.96 (5.41, 6.56) |
|  | 9 | 2291 | 1445 | 63.1% | 1.01% | 5.35 (4.83, 5.92) |
|  | 10 | 2047 | 1297 | 63.4% | 1.07% | 4.9 (4.4, 5.46) |
|  | 11 | 1825 | 1166 | 63.9% | 1.12% | 4.6 (4.1, 5.16) |
|  | 12 | 1655 | 1069 | 64.6% | 1.18% | 4.44 (3.92, 5.01) |
|  | 13 | 1477 | 959 | 64.9% | 1.24% | 4.21 (3.7, 4.79) |
|  | 14 | 1305 | 846 | 64.8% | 1.32% | 3.8 (3.31, 4.36) |
|  | 15 | 1132 | 744 | 65.7% | 1.41% | 3.58 (3.08, 4.15) |
|  | 16 | 1015 | 663 | 65.3% | 1.49% | 3.2 (2.73, 3.75) |
|  | 17 | 889 | 595 | 66.9% | 1.58% | 3.22 (2.72, 3.82) |
|  | 18 | 783 | 524 | 66.9% | 1.68% | 3.05 (2.55, 3.66) |
|  | 19 | 717 | 480 | 66.9% | 1.76% | 2.88 (2.38, 3.49) |
|  | 20 | 649 | 435 | 67.0% | 1.85% | 2.75 (2.25, 3.36) |
|  | 21 | 586 | 399 | 68.1% | 1.93% | 2.75 (2.21, 3.4) |
|  | 22 | 522 | 358 | 68.6% | 2.03% | 2.6 (2.07, 3.26) |
|  | 23 | 476 | 326 | 68.5% | 2.13% | 2.52 (1.98, 3.2) |
|  | 24 | 434 | 300 | 69.1% | 2.22% | 2.54 (1.98, 3.26) |
|  | 25 | 397 | 275 | 69.3% | 2.32% | 2.44 (1.87, 3.17) |
|  | 26 | 361 | 252 | 69.8% | 2.42% | 2.44 (1.85, 3.22) |
|  | 27 | 335 | 236 | 70.4% | 2.49% | 2.42 (1.81, 3.25) |
|  | 28 | 304 | 216 | 71.1% | 2.60% | 2.41 (1.77, 3.29) |
| **Moderate (33-66%)** | |  |  |  |  |  |
|  | 1 | 11974 | 5488 | 45.8% | 0.46% | 13.3 (12.7, 13.9) |
|  | 2 | 10709 | 4698 | 43.9% | 0.48% | 8.76 (8.36, 9.17) |
|  | 3 | 9406 | 3987 | 42.4% | 0.51% | 6.39 (6.08, 6.72) |
|  | 4 | 8255 | 3466 | 42.0% | 0.54% | 5.29 (5.01, 5.58) |
|  | 5 | 7192 | 3028 | 42.1% | 0.58% | 4.56 (4.31, 4.84) |
|  | 6 | 6291 | 2666 | 42.4% | 0.62% | 4 (3.76, 4.26) |
|  | 7 | 5476 | 2374 | 43.4% | 0.67% | 3.61 (3.38, 3.86) |
|  | 8 | 4760 | 2091 | 43.9% | 0.72% | 3.26 (3.03, 3.5) |
|  | 9 | 4150 | 1863 | 44.9% | 0.77% | 3.03 (2.8, 3.27) |
|  | 10 | 3619 | 1671 | 46.2% | 0.83% | 2.89 (2.67, 3.14) |
|  | 11 | 3182 | 1493 | 46.9% | 0.88% | 2.78 (2.55, 3.03) |
|  | 12 | 2792 | 1331 | 47.7% | 0.95% | 2.69 (2.45, 2.95) |
|  | 13 | 2442 | 1172 | 48.0% | 1.01% | 2.54 (2.3, 2.8) |
|  | 14 | 2114 | 1032 | 48.8% | 1.09% | 2.41 (2.17, 2.68) |
|  | 15 | 1800 | 903 | 50.2% | 1.18% | 2.32 (2.07, 2.61) |
|  | 16 | 1558 | 801 | 51.4% | 1.27% | 2.26 (2, 2.55) |
|  | 17 | 1378 | 724 | 52.5% | 1.35% | 2.24 (1.97, 2.55) |
|  | 18 | 1220 | 646 | 53.0% | 1.43% | 2.17 (1.89, 2.49) |
|  | 19 | 1115 | 598 | 53.6% | 1.49% | 2.13 (1.84, 2.46) |
|  | 20 | 996 | 544 | 54.6% | 1.58% | 2.1 (1.8, 2.45) |
|  | 21 | 891 | 497 | 55.8% | 1.66% | 2.1 (1.79, 2.47) |
|  | 22 | 805 | 455 | 56.5% | 1.75% | 2.04 (1.72, 2.42) |
|  | 23 | 733 | 412 | 56.2% | 1.83% | 1.98 (1.66, 2.37) |
|  | 24 | 678 | 376 | 55.5% | 1.91% | 1.86 (1.54, 2.23) |
|  | 25 | 617 | 340 | 55.1% | 2.00% | 1.73 (1.42, 2.1) |
|  | 26 | 564 | 311 | 55.1% | 2.10% | 1.68 (1.37, 2.06) |
|  | 27 | 517 | 288 | 55.7% | 2.19% | 1.67 (1.35, 2.07) |
|  | 28 | 483 | 273 | 56.5% | 2.26% | 1.68 (1.35, 2.1) |
| **Low (<33%)** |  |  |  |  |  |  |
|  | 1 | 264179 | 10325 | 3.9% | 0.04% | Ref. |
|  | 2 | 136741 | 8613 | 6.3% | 0.07% | Ref. |
|  | 3 | 89052 | 7138 | 8.0% | 0.09% | Ref. |
|  | 4 | 63152 | 6020 | 9.5% | 0.12% | Ref. |
|  | 5 | 46214 | 5158 | 11.2% | 0.15% | Ref. |
|  | 6 | 35191 | 4510 | 12.8% | 0.18% | Ref. |
|  | 7 | 27340 | 3995 | 14.6% | 0.21% | Ref. |
|  | 8 | 21527 | 3527 | 16.4% | 0.25% | Ref. |
|  | 9 | 17645 | 3180 | 18.0% | 0.29% | Ref. |
|  | 10 | 14811 | 2874 | 19.4% | 0.32% | Ref. |
|  | 11 | 12629 | 2594 | 20.5% | 0.36% | Ref. |
|  | 12 | 10865 | 2349 | 21.6% | 0.39% | Ref. |
|  | 13 | 9418 | 2147 | 22.8% | 0.43% | Ref. |
|  | 14 | 8088 | 1965 | 24.3% | 0.48% | Ref. |
|  | 15 | 6710 | 1772 | 26.4% | 0.54% | Ref. |
|  | 16 | 5728 | 1615 | 28.2% | 0.59% | Ref. |
|  | 17 | 5049 | 1482 | 29.4% | 0.64% | Ref. |
|  | 18 | 4484 | 1364 | 30.4% | 0.69% | Ref. |
|  | 19 | 4028 | 1262 | 31.3% | 0.73% | Ref. |
|  | 20 | 3616 | 1171 | 32.4% | 0.78% | Ref. |
|  | 21 | 3232 | 1095 | 33.9% | 0.83% | Ref. |
|  | 22 | 2926 | 1026 | 35.1% | 0.88% | Ref. |
|  | 23 | 2651 | 945 | 35.6% | 0.93% | Ref. |
|  | 24 | 2445 | 893 | 36.5% | 0.97% | Ref. |
|  | 25 | 2243 | 842 | 37.5% | 1.02% | Ref. |
|  | 26 | 2067 | 792 | 38.3% | 1.07% | Ref. |
|  | 27 | 1915 | 746 | 39.0% | 1.11% | Ref. |
|  | 28 | 1764 | 702 | 39.8% | 1.17% | Ref. |

Probability of death is based on their ICU admission total predicted risk of death from a logistic regression including both antecedent characteristics and acute illness component. Odds ratios and their 95% CI are odds for in-hospital death for patients with high and moderate probability of death on admission versus those with low probability of death of those still in an ICU on a given day. This statistic is adjusted for differences between groups in antecedent characteristics component.

**Supplementary Table 3.** Regression coefficients for antecedent characteristics component.

|  | Survived | Died | Odds ratio | P |
| --- | --- | --- | --- | --- |
| Variables | N=262,260 | N=23,307 | (95% CI) | value |
| Age category |  |  |  |  |
| <45 | 24,785 (9.5%) | 1,283 (5.5%) | Ref. |  |
| ≥45 | 43,773 (16.7%) | 3,013 (12.9%) | 1.31 (1.22, 1.40) | <0.001 |
| ≥60 | 22,510 (8.6%) | 1,622 (7.0%) | 1.40 (1.29, 1.51) | <0.001 |
| ≥65 | 33,399 (12.7%) | 2,504 (10.7%) | 1.51 (1.40, 1.62) | <0.001 |
| ≥70 | 44,565 (17.0%) | 3,578 (15.4%) | 1.69 (1.58, 1.81) | <0.001 |
| ≥75 | 71,090 (27.1%) | 7,566 (32.5%) | 2.31 (2.17, 2.46) | <0.001 |
| ≥85 | 22,138 (8.4%) | 3,741 (16.1%) | 3.53 (3.30, 3.79) | <0.001 |
| Male | 160,031 (61.0%) | 14,957 (64.2%) | 1.16 (1.13, 1.20) | <0.001 |
| Comorbidities |  |  |  |  |
| AIDS | 132 (0.1%) | 30 (0.1%) | 0.60 (0.36, 1.02) | 0.058 |
| Cardiovascular disease | 3,774 (1.4%) | 719 (3.1%) | 1.75 (1.60, 1.91) | <0.001 |
| Respiratory disease | 2,256 (0.9%) | 764 (3.3%) | 2.98 (2.72, 3.26) | <0.001 |
| Hepatic failure | 889 (0.3%) | 482 (2.1%) | 2.75 (2.37, 3.20) | <0.001 |
| Cirrhosis | 2,559 (1.0%) | 858 (3.7%) | 2.57 (2.31, 2.86) | <0.001 |
| Leukemia | 870 (0.3%) | 761 (3.3%) | 7.78 (6.97, 8.68) | <0.001 |
| Lymphoma | 1,215 (0.5%) | 596 (2.6%) | 4.38 (3.93, 4.89) | <0.001 |
| Metastatic cancer | 9,530 (3.6%) | 1,503 (6.4%) | 1.79 (1.68, 1.90) | <0.001 |
| Immunosuppression | 14,289 (5.4%) | 3,085 (13.2%) | 2.39 (2.27, 2.51) | <0.001 |
| Renal dysfunction | 11,710 (4.5%) | 2,200 (9.4%) | 2.49 (2.36, 2.62) | <0.001 |
| Hospital type |  |  |  |  |
| Public Hospital | 38,773 (14.8%) | 4,504 (19.3%) | Ref. |  |
| Public University | 11,586 (4.4%) | 1,286 (5.5%) | 0.83 (0.78, 0.89) | <0.001 |
| Municipal Hospital | 26,473 (10.1%) | 2,671 (11.5%) | 0.88 (0.83, 0.93) | <0.001 |
| National University | 71,265 (27.2%) | 4,392 (18.8%) | 0.65 (0.62, 0.68) | <0.001 |
| National Hospital | 11,212 (4.3%) | 1,442 (6.2%) | 1.09 (1.02, 1.17) | 0.009 |
| Private University | 61,127 (23.3%) | 4,673 (20.0%) | 0.95 (0.90, 1.00) | 0.046 |
| Private Hospital | 41,824 (15.9%) | 4,339 (18.6%) | 1.01 (0.96, 1.06) | 0.725 |
| ICU volume per year |  |  |  |  |
| 1-499 | 33,485 (12.8%) | 5,205 (22.3%) | Ref. |  |
| 500-999 | 122,368 (46.7%) | 11,883 (51.0%) | 0.63 (0.61, 0.66) | <0.001 |
| 1000-1499 | 66,278 (25.3%) | 4,431 (19.0%) | 0.49 (0.47, 0.52) | <0.001 |
| 1500-1999 | 38,104 (14.5%) | 1,676 (7.2%) | 0.37 (0.35, 0.40) | <0.001 |
| 2000-2500 | 2,025 (0.8%) | 112 (0.5%) | 0.30 (0.25, 0.37) | <0.001 |
| Fiscal year |  |  |  |  |
| 2015 | 3,491 (1.3%) | 299 (1.3%) | Ref. |  |
| 2016 | 11,052 (4.2%) | 828 (3.6%) | 0.92 (0.80, 1.06) | 0.251 |
| 2017 | 20,240 (7.7%) | 1,602 (6.9%) | 1.02 (0.89, 1.17) | 0.735 |
| 2018 | 28,154 (10.7%) | 2,436 (10.5%) | 1.09 (0.96, 1.25) | 0.185 |
| 2019 | 36,615 (14.0%) | 3,188 (13.7%) | 1.12 (0.98, 1.28) | 0.09 |
| 2020 | 41,306 (15.8%) | 3,537 (15.2%) | 1.03 (0.91, 1.18) | 0.63 |
| 2021 | 48,702 (18.6%) | 4,351 (18.7%) | 1.10 (0.97, 1.25) | 0.153 |
| 2022 | 57,655 (22.0%) | 5,555 (23.8%) | 1.15 (1.01, 1.30) | 0.037 |
| 2023 | 15,045 (5.7%) | 1,511 (6.5%) | 1.17 (1.02, 1.35) | 0.028 |
| Month |  |  |  |  |
| January | 21,951 (8.4%) | 2,304 (9.9%) | Ref. |  |
| February | 20,550 (7.8%) | 1,925 (8.3%) | 0.91 (0.85, 0.97) | 0.004 |
| March | 23,027 (8.8%) | 1,958 (8.4%) | 0.85 (0.79, 0.91) | <0.001 |
| April | 21,647 (8.3%) | 1,924 (8.3%) | 0.89 (0.83, 0.95) | 0.001 |
| May | 20,384 (7.8%) | 1,804 (7.7%) | 0.87 (0.81, 0.93) | <0.001 |
| June | 22,232 (8.5%) | 1,775 (7.6%) | 0.83 (0.77, 0.89) | <0.001 |
| July | 22,340 (8.5%) | 1,732 (7.4%) | 0.78 (0.73, 0.84) | <0.001 |
| August | 21,965 (8.4%) | 1,921 (8.2%) | 0.91 (0.85, 0.97) | 0.006 |
| September | 20,712 (7.9%) | 1,875 (8.0%) | 0.93 (0.86, 0.99) | 0.029 |
| October | 22,705 (8.7%) | 1,952 (8.4%) | 0.86 (0.81, 0.92) | <0.001 |
| November | 22,301 (8.5%) | 1,977 (8.5%) | 0.89 (0.83, 0.95) | 0.001 |
| December | 22,446 (8.6%) | 2,160 (9.3%) | 0.96 (0.89, 1.02) | 0.181 |
| Day |  |  |  |  |
| Sunday | 11,639 (4.4%) | 2,692 (11.6%) | Ref. |  |
| Monday | 49,375 (18.8%) | 3,804 (16.3%) | 0.49 (0.46, 0.51) | <0.001 |
| Tuesday | 49,629 (18.9%) | 3,578 (15.4%) | 0.45 (0.43, 0.48) | <0.001 |
| Wednesday | 47,916 (18.3%) | 3,508 (15.1%) | 0.46 (0.44, 0.49) | <0.001 |
| Thursday | 46,710 (17.8%) | 3,484 (14.9%) | 0.47 (0.44, 0.49) | <0.001 |
| Friday | 42,806 (16.3%) | 3,450 (14.8%) | 0.50 (0.47, 0.53) | <0.001 |
| Saturday | 14,185 (5.4%) | 2,791 (12.0%) | 0.90 (0.85, 0.96) | 0.001 |
| Hour |  |  |  |  |
| 0 | 5,110 (1.9%) | 797 (3.4%) | Ref. |  |
| 1 | 4,361 (1.7%) | 719 (3.1%) | 1.06 (0.95, 1.19) | 0.306 |
| 2 | 3,512 (1.3%) | 609 (2.6%) | 1.08 (0.96, 1.22) | 0.205 |
| 3 | 2,823 (1.1%) | 539 (2.3%) | 1.17 (1.03, 1.33) | 0.013 |
| 4 | 2,426 (0.9%) | 448 (1.9%) | 1.12 (0.99, 1.28) | 0.082 |
| 5 | 1,927 (0.7%) | 422 (1.8%) | 1.28 (1.12, 1.47) | <0.001 |
| 6 | 1,748 (0.7%) | 376 (1.6%) | 1.25 (1.08, 1.44) | 0.002 |
| 7 | 1,723 (0.7%) | 413 (1.8%) | 1.41 (1.23, 1.62) | <0.001 |
| 8 | 1,858 (0.7%) | 475 (2.0%) | 1.54 (1.35, 1.76) | <0.001 |
| 9 | 2,733 (1.0%) | 721 (3.1%) | 1.55 (1.38, 1.74) | <0.001 |
| 10 | 5,590 (2.1%) | 1,002 (4.3%) | 1.15 (1.04, 1.28) | 0.008 |
| 11 | 11,570 (4.4%) | 1,278 (5.5%) | 0.79 (0.72, 0.88) | <0.001 |
| 12 | 16,582 (6.3%) | 1,173 (5.0%) | 0.55 (0.50, 0.61) | <0.001 |
| 13 | 21,025 (8.0%) | 1,400 (6.0%) | 0.54 (0.49, 0.59) | <0.001 |
| 14 | 25,105 (9.6%) | 1,574 (6.8%) | 0.51 (0.46, 0.56) | <0.001 |
| 15 | 26,708 (10.2%) | 1,492 (6.4%) | 0.45 (0.41, 0.50) | <0.001 |
| 16 | 26,784 (10.2%) | 1,434 (6.2%) | 0.43 (0.39, 0.48) | <0.001 |
| 17 | 24,894 (9.5%) | 1,512 (6.5%) | 0.49 (0.45, 0.54) | <0.001 |
| 18 | 20,862 (8.0%) | 1,336 (5.7%) | 0.52 (0.47, 0.57) | <0.001 |
| 19 | 16,997 (6.5%) | 1,299 (5.6%) | 0.61 (0.55, 0.67) | <0.001 |
| 20 | 13,149 (5.0%) | 1,190 (5.1%) | 0.70 (0.63, 0.77) | <0.001 |
| 21 | 10,018 (3.8%) | 1,099 (4.7%) | 0.80 (0.72, 0.89) | <0.001 |
| 22 | 7,917 (3.0%) | 1,042 (4.5%) | 0.92 (0.83, 1.02) | 0.101 |
| 23 | 6,838 (2.6%) | 957 (4.1%) | 0.94 (0.85, 1.04) | 0.246 |

Due to the large sample size, many predictors achieved statistical significance. Readers are advised to interpret p-values with caution and focus on the relative magnitude of effect sizes (e.g., odds ratios) for clinical relevance.

AIDS=acquired immuno deficiency syndrome, ICU=intensive care unit.

**Supplementary Table 4.** Regression coefficients for acute illness component.

|  | Survived | Died | Odds ratio | P |
| --- | --- | --- | --- | --- |
| Variables | N=262,260 | N=23,307 | (95% CI) | value |
| APACHE III diagnoses |  |  |  |  |
| Others | 590 (0.2%) | 22 (0.1%) | Ref. |  |
| 101 | 496 (0.2%) | 262 (1.1%) | 0.71 (0.42, 1.21) | 0.211 |
| 102 | 2,960 (1.1%) | 3,903 (16.7%) | 0.34 (0.21, 0.57) | <0.001 |
| 103 | 3,065 (1.2%) | 355 (1.5%) | 0.57 (0.34, 0.96) | 0.033 |
| 104 | 6,132 (2.3%) | 837 (3.6%) | 0.39 (0.24, 0.64) | <0.001 |
| 105 | 344 (0.1%) | 56 (0.2%) | 0.48 (0.27, 0.88) | 0.018 |
| 106 | 1,228 (0.5%) | 93 (0.4%) | 0.21 (0.12, 0.37) | <0.001 |
| 107 | 7,842 (3.0%) | 588 (2.5%) | 0.37 (0.22, 0.61) | <0.001 |
| 108 | 38 (0.0%) | 1 (0.0%) | 0.07 (0.01, 0.57) | 0.013 |
| 109 | 1,871 (0.7%) | 277 (1.2%) | 0.34 (0.20, 0.57) | <0.001 |
| 110 | 339 (0.1%) | 53 (0.2%) | 0.45 (0.25, 0.82) | 0.009 |
| 111 | 1,064 (0.4%) | 26 (0.1%) | 0.12 (0.06, 0.23) | <0.001 |
| 201 | 1,578 (0.6%) | 580 (2.5%) | 0.36 (0.22, 0.60) | <0.001 |
| 202 | 170 (0.1%) | 88 (0.4%) | 1.48 (0.83, 2.64) | 0.189 |
| 203 | 98 (0.0%) | 49 (0.2%) | 0.28 (0.15, 0.55) | <0.001 |
| 204 | 406 (0.2%) | 329 (1.4%) | 1.10 (0.65, 1.86) | 0.726 |
| 206 | 353 (0.1%) | 110 (0.5%) | 0.73 (0.42, 1.26) | 0.255 |
| 207 | 519 (0.2%) | 66 (0.3%) | 0.37 (0.21, 0.67) | 0.001 |
| 208 | 806 (0.3%) | 138 (0.6%) | 0.27 (0.16, 0.46) | <0.001 |
| 209 | 192 (0.1%) | 8 (0.0%) | 0.11 (0.05, 0.29) | <0.001 |
| 210 | 101 (0.0%) | 112 (0.5%) | 2.16 (1.20, 3.87) | 0.01 |
| 211 | 2,110 (0.8%) | 1,000 (4.3%) | 0.99 (0.60, 1.63) | 0.96 |
| 212 | 1,981 (0.8%) | 938 (4.0%) | 0.61 (0.37, 1.01) | 0.055 |
| 213 | 2,614 (1.0%) | 635 (2.7%) | 0.70 (0.42, 1.17) | 0.173 |
| 301 | 221 (0.1%) | 280 (1.2%) | 0.74 (0.43, 1.28) | 0.289 |
| 303 | 336 (0.1%) | 90 (0.4%) | 0.30 (0.17, 0.54) | <0.001 |
| 305 | 1,164 (0.4%) | 242 (1.0%) | 0.28 (0.17, 0.48) | <0.001 |
| 306 | 486 (0.2%) | 70 (0.3%) | 0.28 (0.16, 0.50) | <0.001 |
| 307 | 390 (0.1%) | 102 (0.4%) | 0.37 (0.21, 0.66) | 0.001 |
| 308 | 198 (0.1%) | 104 (0.4%) | 0.51 (0.29, 0.91) | 0.023 |
| 309 | 223 (0.1%) | 81 (0.3%) | 0.37 (0.20, 0.66) | 0.001 |
| 310 | 130 (0.0%) | 132 (0.6%) | 0.75 (0.41, 1.37) | 0.353 |
| 311 | 509 (0.2%) | 127 (0.5%) | 0.39 (0.23, 0.68) | 0.001 |
| 312 | 255 (0.1%) | 88 (0.4%) | 0.76 (0.43, 1.36) | 0.359 |
| 313 | 1,354 (0.5%) | 344 (1.5%) | 0.24 (0.15, 0.41) | <0.001 |
| 401 | 1,115 (0.4%) | 375 (1.6%) | 0.84 (0.50, 1.41) | 0.513 |
| 402 | 737 (0.3%) | 249 (1.1%) | 0.99 (0.59, 1.68) | 0.979 |
| 403 | 2,083 (0.8%) | 253 (1.1%) | 0.53 (0.31, 0.88) | 0.015 |
| 404 | 273 (0.1%) | 76 (0.3%) | 0.26 (0.14, 0.46) | <0.001 |
| 405 | 79 (0.0%) | 26 (0.1%) | 0.98 (0.47, 2.03) | 0.957 |
| 406 | 261 (0.1%) | 27 (0.1%) | 0.23 (0.12, 0.44) | <0.001 |
| 407 | 1,748 (0.7%) | 126 (0.5%) | 0.10 (0.06, 0.18) | <0.001 |
| 408 | 322 (0.1%) | 36 (0.2%) | 0.25 (0.13, 0.47) | <0.001 |
| 409 | 167 (0.1%) | 36 (0.2%) | 0.63 (0.33, 1.21) | 0.166 |
| 410 | 616 (0.2%) | 119 (0.5%) | 0.17 (0.10, 0.30) | <0.001 |
| 501 | 468 (0.2%) | 138 (0.6%) | 0.32 (0.18, 0.54) | <0.001 |
| 502 | 433 (0.2%) | 56 (0.2%) | 0.18 (0.10, 0.33) | <0.001 |
| 503 | 1,486 (0.6%) | 1,082 (4.6%) | 0.40 (0.24, 0.67) | <0.001 |
| 504 | 1,172 (0.4%) | 241 (1.0%) | 0.19 (0.11, 0.33) | <0.001 |
| 601 | 1,684 (0.6%) | 241 (1.0%) | 0.46 (0.27, 0.77) | 0.003 |
| 602 | 1,580 (0.6%) | 75 (0.3%) | 0.18 (0.11, 0.32) | <0.001 |
| 603 | 362 (0.1%) | 99 (0.4%) | 0.64 (0.37, 1.14) | 0.129 |
| 604 | 139 (0.1%) | 16 (0.1%) | 0.27 (0.12, 0.61) | 0.001 |
| 605 | 130 (0.0%) | 19 (0.1%) | 0.85 (0.41, 1.75) | 0.658 |
| 701 | 527 (0.2%) | 101 (0.4%) | 0.17 (0.10, 0.29) | <0.001 |
| 702 | 891 (0.3%) | 37 (0.2%) | 0.06 (0.03, 0.11) | <0.001 |
| 703 | 1,495 (0.6%) | 50 (0.2%) | 0.07 (0.04, 0.13) | <0.001 |
| 704 | 2,461 (0.9%) | 280 (1.2%) | 0.16 (0.09, 0.26) | <0.001 |
| 801 | 215 (0.1%) | 147 (0.6%) | 0.67 (0.38, 1.17) | 0.158 |
| 802 | 309 (0.1%) | 353 (1.5%) | 1.09 (0.64, 1.86) | 0.75 |
| 901 | 937 (0.4%) | 181 (0.8%) | 0.26 (0.16, 0.45) | <0.001 |
| 902 | 25 (0.0%) | 1 (0.0%) | 0.08 (0.01, 0.92) | 0.042 |
| 903 | 265 (0.1%) | 0 (0.0%) | 1.00 (0.00, 0.00) | <0.001 |
| 1002 | 580 (0.2%) | 95 (0.4%) | 0.25 (0.14, 0.44) | <0.001 |
| 1101 | 225 (0.1%) | 79 (0.3%) | 0.45 (0.25, 0.81) | 0.007 |
| 1102 | 511 (0.2%) | 205 (0.9%) | 0.37 (0.21, 0.63) | <0.001 |
| 1202 | 1,313 (0.5%) | 86 (0.4%) | 0.56 (0.32, 1.01) | 0.052 |
| 1203 | 1,167 (0.4%) | 51 (0.2%) | 0.55 (0.30, 1.01) | 0.052 |
| 1204 | 9,212 (3.5%) | 244 (1.0%) | 0.50 (0.29, 0.87) | 0.013 |
| 1205 | 810 (0.3%) | 9 (0.0%) | 0.43 (0.18, 0.99) | 0.048 |
| 1206 | 16,695 (6.4%) | 335 (1.4%) | 0.28 (0.17, 0.49) | <0.001 |
| 1207 | 8,700 (3.3%) | 163 (0.7%) | 0.19 (0.11, 0.32) | <0.001 |
| 1208 | 11,242 (4.3%) | 339 (1.5%) | 0.37 (0.22, 0.63) | <0.001 |
| 1209 | 3,636 (1.4%) | 374 (1.6%) | 0.47 (0.27, 0.80) | 0.005 |
| 1210 | 1,299 (0.5%) | 229 (1.0%) | 0.54 (0.31, 0.94) | 0.029 |
| 1211 | 206 (0.1%) | 11 (0.0%) | 0.83 (0.37, 1.89) | 0.656 |
| 1212 | 2,088 (0.8%) | 98 (0.4%) | 0.44 (0.25, 0.77) | 0.004 |
| 1213 | 6,739 (2.6%) | 120 (0.5%) | 0.44 (0.25, 0.76) | 0.004 |
| 1214 | 1,136 (0.4%) | 3 (0.0%) | 0.08 (0.02, 0.29) | <0.001 |
| 1301 | 622 (0.2%) | 44 (0.2%) | 0.54 (0.29, 1.01) | 0.054 |
| 1302 | 16,160 (6.2%) | 120 (0.5%) | 0.35 (0.20, 0.61) | <0.001 |
| 1303 | 5,961 (2.3%) | 71 (0.3%) | 0.29 (0.16, 0.52) | <0.001 |
| 1304 | 2,807 (1.1%) | 97 (0.4%) | 0.55 (0.31, 0.97) | 0.04 |
| 1401 | 4,269 (1.6%) | 689 (3.0%) | 0.47 (0.27, 0.80) | 0.005 |
| 1403 | 281 (0.1%) | 31 (0.1%) | 0.34 (0.17, 0.66) | 0.002 |
| 1404 | 2,373 (0.9%) | 182 (0.8%) | 0.35 (0.20, 0.60) | <0.001 |
| 1405 | 36,264 (13.8%) | 452 (1.9%) | 0.32 (0.19, 0.54) | <0.001 |
| 1406 | 1,136 (0.4%) | 43 (0.2%) | 0.18 (0.10, 0.34) | <0.001 |
| 1407 | 243 (0.1%) | 20 (0.1%) | 0.31 (0.15, 0.63) | 0.001 |
| 1408 | 2,300 (0.9%) | 99 (0.4%) | 0.36 (0.20, 0.63) | <0.001 |
| 1409 | 327 (0.1%) | 18 (0.1%) | 0.28 (0.13, 0.60) | 0.001 |
| 1410 | 688 (0.3%) | 200 (0.9%) | 0.63 (0.36, 1.09) | 0.1 |
| 1411 | 54 (0.0%) | 3 (0.0%) | 0.35 (0.08, 1.55) | 0.166 |
| 1412 | 534 (0.2%) | 91 (0.4%) | 0.44 (0.25, 0.79) | 0.006 |
| 1413 | 549 (0.2%) | 24 (0.1%) | 0.25 (0.12, 0.49) | <0.001 |
| 1501 | 1,805 (0.7%) | 295 (1.3%) | 0.64 (0.37, 1.10) | 0.107 |
| 1502 | 723 (0.3%) | 143 (0.6%) | 0.63 (0.36, 1.11) | 0.109 |
| 1503 | 2,422 (0.9%) | 287 (1.2%) | 0.72 (0.42, 1.24) | 0.236 |
| 1504 | 5,600 (2.1%) | 62 (0.3%) | 0.28 (0.16, 0.50) | <0.001 |
| 1505 | 8,760 (3.3%) | 139 (0.6%) | 0.59 (0.34, 1.02) | 0.058 |
| 1506 | 5,591 (2.1%) | 85 (0.4%) | 0.46 (0.26, 0.82) | 0.008 |
| 1507 | 1,249 (0.5%) | 2 (0.0%) | 0.08 (0.02, 0.37) | 0.001 |
| 1508 | 3,108 (1.2%) | 14 (0.1%) | 0.26 (0.13, 0.56) | <0.001 |
| 1509 | 1,011 (0.4%) | 159 (0.7%) | 0.96 (0.55, 1.67) | 0.876 |
| 1601 | 901 (0.3%) | 241 (1.0%) | 0.88 (0.51, 1.53) | 0.658 |
| 1602 | 1,560 (0.6%) | 89 (0.4%) | 0.28 (0.16, 0.50) | <0.001 |
| 1603 | 79 (0.0%) | 19 (0.1%) | 2.30 (1.05, 5.04) | 0.037 |
| 1604 | 172 (0.1%) | 7 (0.0%) | 0.26 (0.10, 0.68) | 0.006 |
| 1605 | 201 (0.1%) | 14 (0.1%) | 0.61 (0.28, 1.33) | 0.213 |
| 1701 | 7,404 (2.8%) | 37 (0.2%) | 0.15 (0.08, 0.28) | <0.001 |
| 1703 | 976 (0.4%) | 54 (0.2%) | 0.27 (0.15, 0.50) | <0.001 |
| 1704 | 973 (0.4%) | 1 (0.0%) | 0.02 (0.00, 0.12) | <0.001 |
| 1705 | 74 (0.0%) | 2 (0.0%) | 0.39 (0.08, 1.85) | 0.239 |
| 1801 | 3,159 (1.2%) | 17 (0.1%) | 0.16 (0.08, 0.32) | <0.001 |
| 1802 | 924 (0.4%) | 4 (0.0%) | 0.03 (0.01, 0.11) | <0.001 |
| 1803 | 1,170 (0.4%) | 30 (0.1%) | 0.42 (0.22, 0.80) | 0.008 |
| 1902 | 5,601 (2.1%) | 170 (0.7%) | 0.41 (0.24, 0.71) | 0.002 |
| 1903 | 2,089 (0.8%) | 38 (0.2%) | 0.35 (0.19, 0.65) | 0.001 |
| 1904 | 877 (0.3%) | 123 (0.5%) | 0.41 (0.23, 0.73) | 0.002 |
| 2101 | 53 (0.0%) | 12 (0.1%) | 2.52 (1.01, 6.29) | 0.049 |
| 2201 | 1,508 (0.6%) | 12 (0.1%) | 0.25 (0.11, 0.55) | 0.001 |
| Physiology score |  |  |  |  |
| Heart rate | 2.3 (3.5) | 6.1 (5.1) | 1.05 (1.05, 1.06) | <0.001 |
| Mean arterial pressure | 9.2 (4.5) | 14.1 (6.3) | 1.06 (1.06, 1.07) | <0.001 |
| Temperature | 0.8 (2.5) | 2.6 (5.1) | 1.03 (1.02, 1.03) | <0.001 |
| Respiratory rate | 6.5 (3.0) | 8.4 (4.7) | 1.05 (1.05, 1.06) | <0.001 |
| PaO2 | 3.9 (5.2) | 6.3 (5.3) | 1.00 (1.00, 1.01) | 0.064 |
| Haematocrit | 2.8 (0.8) | 2.7 (0.9) | 0.93 (0.91, 0.95) | <0.001 |
| White cell count | 0.3 (1.6) | 1.7 (4.3) | 1.05 (1.04, 1.05) | <0.001 |
| Creatinine | 1.1 (2.5) | 3.6 (4.0) | 1.02 (1.02, 1.03) | <0.001 |
| Urine output | 4.6 (3.9) | 8.4 (5.5) | 1.08 (1.08, 1.09) | <0.001 |
| Urea | 3.0 (3.8) | 6.6 (4.3) | 1.07 (1.06, 1.07) | <0.001 |
| Sodium | 0.6 (0.9) | 1.0 (1.2) | 1.11 (1.09, 1.12) | <0.001 |
| Albumin | 1.4 (3.0) | 3.6 (4.4) | 1.05 (1.05, 1.06) | <0.001 |
| Bilirubin | 0.6 (1.8) | 1.4 (3.3) | 1.09 (1.08, 1.10) | <0.001 |
| Glucose | 0.8 (1.6) | 2.0 (2.6) | 1.07 (1.06, 1.08) | <0.001 |
| pH | 2.7 (4.0) | 5.0 (4.5) | 1.02 (1.02, 1.03) | <0.001 |
| GCS | 2.5 (8.5) | 20.2 (21.1) | 1.04 (1.04, 1.04) | <0.001 |
| Type of ICU admission |  |  |  |  |
| Elective surgery | 164,814 (62.8%) | 2,201 (9.4%) | Ref. |  |
| Urgent surgery | 32,433 (12.4%) | 4,095 (17.6%) | 3.26 (2.96, 3.60) | <0.001 |
| Medical | 65,013 (24.8%) | 17,011 (73.0%) | 4.60 (3.11, 6.82) | <0.001 |
| Source of ICU admission |  |  |  |  |
| Emergency department | 190,971 (72.8%) | 5,446 (23.4%) | Ref. |  |
| Operation room | 51,479 (19.6%) | 9,546 (41.0%) | 1.04 (0.95, 1.14) | 0.442 |
| General ward | 16,535 (6.3%) | 7,124 (30.6%) | 1.20 (1.08, 1.32) | <0.001 |
| Other | 3,275 (1.2%) | 1,191 (5.1%) | 1.22 (1.08, 1.38) | 0.001 |
| Pre-ICU hospital length of stay |  |  |  |  |
| On the day of admission | 66,031 (25.2%) | 11,456 (49.2%) | Ref. |  |
| Day 2 | 52,712 (20.1%) | 2,576 (11.1%) | 0.99 (0.93, 1.06) | 0.844 |
| Day 3 | 40,241 (15.3%) | 982 (4.2%) | 1.00 (0.91, 1.10) | 0.969 |
| Day 4-6 | 51,791 (19.7%) | 1,917 (8.2%) | 1.32 (1.22, 1.43) | <0.001 |
| Day ≥7 | 51,485 (19.6%) | 6,376 (27.4%) | 2.16 (2.02, 2.31) | <0.001 |
| IMV within first 24 hour | 87,767 (33.5%) | 15,198 (65.2%) | 1.15 (1.10, 1.20) | <0.001 |
| Medical emergency team call |  |  |  |  |
| None | 258,414 (98.5%) | 20,684 (88.7%) | Ref. |  |
| RRT/MET | 2,673 (1.0%) | 1,445 (6.2%) | 0.98 (0.89, 1.07) | 0.595 |
| Code blue | 1,173 (0.4%) | 1,178 (5.1%) | 0.77 (0.68, 0.88) | <0.001 |
| Cardiac arrest | 3,927 (1.5%) | 4,587 (19.7%) | 1.78 (1.57, 2.03) | <0.001 |

Due to the large sample size, many predictors achieved statistical significance. Readers are advised to interpret p-values with caution and focus on the relative magnitude of effect sizes (e.g., odds ratios) for clinical relevance.

APACHE=Acute Physiology and Chronic Health Evaluation, GCS=Glasgow coma scale, ICU=Intensive care unit, IMV=Invasive mechanical ventilation, RRT/MET=Renal replacement therapy/Medical emergency team.

**Supplementary Figure 1.** Patient flow chart.

ICU=intensive care unit, JIPAD=Japanese Intensive Care Patient Database.

**Supplementary Figure 2.** Predictive ability (measured by AUROC) for in-hospital mortality in the models of antecedent characteristics and acute illness component in the subgroup analyses for age and type of ICU admission.

AUROC=area under the receiver operating characteristics, CI=confidence interval, ICU=intensive care unit.

**Supplementary Figure 3.** Predictive ability (measured by AUROC) for in-hospital mortality in the models of antecedent characteristics and acute illness component in the subgroup analyses for fiscal year and admission diagnosis.

AUROC=area under the receiver operating characteristics, CI=confidence interval, ICU=intensive care unit.

**Supplementary Figure 4**. Predictive ability (measured by AUROC) for in-hospital mortality in the models of antecedent characteristics and acute illness component in the sensitivity analysis excluding day of the week and time of day from the antecedent characteristics model.

Vertical bars are 95% CIs. The Supplemental Table 3 and 4 contains all characteristics and regression weights (β coefficients from derivation sample).

AUROC=area under the receiver operating characteristics, CI=confidence interval, ICU=intensive care unit.
